# Neutron Source Based on Vacuum Insulated Tandem Accelerator and Lithium Target

**DOI:** 10.3390/biology10050350

**Published:** 2021-04-21

**Authors:** Sergey Taskaev, Evgenii Berendeev, Marina Bikchurina, Timofey Bykov, Dmitrii Kasatov, Iaroslav Kolesnikov, Alexey Koshkarev, Aleksandr Makarov, Georgii Ostreinov, Vyacheslav Porosev, Sergey Savinov, Ivan Shchudlo, Evgeniia Sokolova, Igor Sorokin, Tatiana Sycheva, Gleb Verkhovod

**Affiliations:** 1 Budker Institute of Nuclear Physics, 11 Lavrentiev Ave., 630090 Novosibirsk, Russia; evgeny.berendeev@gmail.com (E.B.); timaisabrony@gmail.com (T.B.); kasatovd@gmail.com (D.K.); katyono@mail.ru (I.K.); alexxmak314@gmail.com (A.M.); wtfsnoo@gmail.com (G.O.); porosev@gmail.com (V.P.); savinov89@gmail.com (S.S.); cshudlo.i.m@gmail.com (I.S.); I.N.Sorokin@inp.nsk.su (I.S.); sychevatatyanav@gmail.com (T.S.); 2 Faculty of Physics, Novosibirsk State University, 2 Pirogov Str., 630090 Novosibirsk, Russia; knkstdor@gmail.com (M.B.); kent_brockman4@mail.ru (A.K.); buiya@bk.ru (E.S.); thevoidscreamer@gmail.com (G.V.)

**Keywords:** boron neutron capture therapy, neutron source, charge particle accelerator, lithium target, neutron detector

## Abstract

**Simple Summary:**

A compact neutron source has been proposed and created at the Budker Institute of Nuclear Physics in Novosibirsk, Russia. The source comprises an original design tandem accelerator, solid lithium target, and a neutron beam shaping assembly. The neutron source is capable of producing the high neutron flux in various energy ranges, from thermal to fast, for boron neutron capture therapy, as well as for other applications. A lot of scientific research has been carried out at the facility, including the study of blistering and its effect on the neutron yield. The Boron Neutron Capture Therapy (BNCT) technique is being tested in in vitro and in vivo studies, and the methods of dosimetry are being developed. It is planned to certify the neutron source next year and conduct clinical trials on it. The neutron source served as a prototype for a facility created for a clinic in Xiamen (China).

**Abstract:**

A compact accelerator-based neutron source has been proposed and created at the Budker Institute of Nuclear Physics in Novosibirsk, Russia. An original design tandem accelerator is used to provide a proton beam. The proton beam energy can be varied within a range of 0.6–2.3 MeV, keeping a high-energy stability of 0.1%. The beam current can also be varied in a wide range (from 0.3 mA to 10 mA) with high current stability (0.4%). In the device, neutron flux is generated as a result of the ^7^Li(p,n)^7^Be threshold reaction. A beam-shaping assembly is applied to convert this flux into a beam of epithermal neutrons with characteristics suitable for BNCT. A lot of scientific research has been carried out at the facility, including the study of blistering and its effect on the neutron yield. The BNCT technique is being tested in in vitro and in vivo studies, and the methods of dosimetry are being developed. It is planned to certify the neutron source next year and conduct clinical trials on it. The neutron source served as a prototype for a facility created for a clinic in Xiamen (China).

## 1. Introduction

The main objective for the accelerator-based Boron Neutron Capture Therapy (BNCT) system is to design a compact neutron source that best satisfies the BNCT requirements [1], namely, a source of beam of epithermal neutrons with minimized fraction of fast and thermal neutrons. Ideally, a neutron source for BNCT should generate a monoenergetic neutron beam with an energy of ~10 keV. Monoenergetic neutron beams with low particle energy are obtained for metrological purposes using the ^7^Li(p,n)^7^Be and ^45^Sc(p,n)^45^Ti reactions [2,3,4]. However, the intensity of these beams is insufficient for clinical BNCT applications. Low intensity is caused by high losses of the entire neutron flux in kinematic collimators or filters used to form the monoenergetic beams.

In order to form the narrowest neutron spectrum within the epithermal energy range, it was proposed to use the ^7^Li(p,n)^7^Be reaction with a high-current, low-energy proton beam for BNCT [5]. Experimental implementation of the proposal required two decades of research, culminating in the creation of a compact reliable neutron source demanded by BNCT and other applications.

In this article, we give a description of the constituent parts of the neutron source, the developed diagnostics, and techniques. When presenting the material, we adhere to the overview presentation of the published materials paying attention to the identified features, and a more detailed description of the results obtained in the last year.

## 2. Materials and Methods

**Figure 1:**
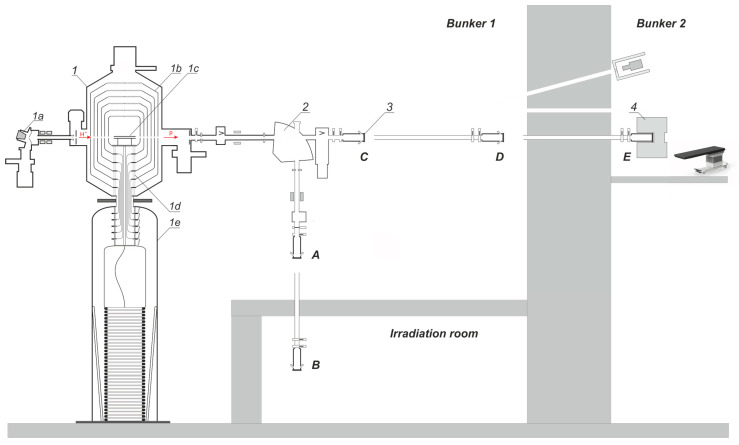
Layout of the experimental facility: *1*—vacuum-insulated tandem accelerator (*1a*—negative ion source, *1b*—intermediate- and high-voltage electrodes, *1c*—gas stripper, *1d*—feedthrough insulator, *1e*—high-voltage power supply), *2*—bending magnet, *3*—lithium target, *4*—beam-shaping assembly. ***A***, ***B***, ***C***, ***D***, ***E***—lithium target placement positions.

Experimental studies were carried out on an accelerator-based neutron source proposed and created at the Budker Institute of Nuclear Physics, Novosibirsk, Russia [6]. The layout of the facility is shown in Figure 1.

The neutron source comprises an original design tandem accelerator, solid lithium target, a neutron beam shaping assembly, and is placed in two bunkers as shown in Figure 1. Each bunker is 10.8 m × 9.1 m and 10 m height, the wall thickness of the bunker is from 1.2 m to 1.3 m, the wall thickness between the bunkers is 1.47 m.

The facility has the ability to place a lithium target in 5 positions; in Figure 1, they are marked as positions *A*, *B*, *C*, *D*, *E*.

With the bending magnet turned on, the proton beam is directed downward. Placing the target at position *A* was the original and commonly used since 2008 for neutron generation and research. In 2020, an irradiation room was built inside bunker 1 with walls 46 cm thick from concrete blocks with boron carbide and a ceiling 8 cm thick made C5 Neutrostop borated polyethylene blocks (KOPOS KOLÍN a.s., Kolín, Czech Republic). In 2021, the lithium target was moved to position *B* to generate powerful neutron fluxes for in vitro and in vivo studies, as well as for the planned fast neutron radiation testing of ITER and CERN materials. The presence of an irradiation room significantly reduces the effect of neutron radiation on the performance of the facility.

With the magnet off, the target is placed in position *C* for measuring the ^7^Li(p,p’ γ)^7^Li reaction cross section and the 478 keV photon yield from a thick lithium target, for measuring the spatial distribution of the lithium layer thickness, in position *D* for boron imaging using the prompt γ-ray spectroscopy with a neutron beam exclusively in the epithermal energy range, and in position *E* for clinical trials.

## 3. Results and Discussion

### 3.1. Vacuum-Insulated Tandem Accelerator

In order to generate a high-current, low-energy proton beam, a DC tandem accelerator is used. The term “tandem” means that the applied DC accelerating voltage is used twice. Negative hydrogen ions are injected to the input of the tandem accelerator, accelerated by a positive potential applied to the central electrode, then stripped to the positive ions, and accelerated again by the same potential. A key advantage of the tandem acceleration concept is to reduce the necessary accelerating voltage by half, which tremendously simplifies electrostatic insulation and consequently reduces the size and cost of the accelerator.

The BINP tandem accelerator, which was named as Vacuum-Insulated Tandem Accelerator (VITA), has a specific design that does not involve accelerating tubes, unlike conventional tandem accelerators. Instead of those, the nested intermediate electrodes (*1b*) fixed at a feedthrough insulator (*1d*) is used, as shown in Figure 1. The advantage of such an arrangement is moving ceramic parts of the feedthrough insulator far enough from the ion beam, thus increasing the high-voltage strength of the accelerating gaps given high ion beam current.

A consequence of this design was also a fast rate of ion acceleration—up to 25 keV/cm.

The proton beam energy can be varied within a range of 0.6–2.3 MeV, keeping a high-energy stability of 0.1%. The beam current can also be varied in a wide range (from 0.3 mA to 10 mA) with high current stability (0.4%). The tandem accelerator is also capable of generating a deuteron beam with similar characteristics.

The details of the studies that made it possible to achieve such parameters are presented in a review article [7] and in recent publications [8,9,10,11].

The potential is supplied for the high-voltage electrode and five intermediate electrodes of the accelerator from a sectioned rectifier through a feedthrough insulator in which a resistive voltage divider is mounted. For the compactness of the accelerator, the average electric field strength on the insulator was chosen to be 14 kV/cm, which is 1.5 times higher than the recommended one. This led to breakdowns along the surface of smooth ceramic insulators with a height of 73 mm, from which the feedthrough insulator was assembled. Such breakdowns occurred approximately once every 10–20 min; they did not lead to a decrease in the electric strength of the accelerator, but required 15 s to restore the parameters of the ion beam. To eliminate breakdowns along the vacuum surface of insulators, the height of individual ceramic insulators was increased from 73 mm to 100 mm in the accelerator for Xiamen BNCT Center. Smooth ceramic insulators were replaced with corrugated insulators of the same height in the accelerator in Novosibirsk [8].

A beam of negative hydrogen ions with an energy of 20–30 keV is injected into the tandem accelerator. A surface plasma source with a normalized emittance of about 0.3 π mm mrad [12] is used at the facility in Novosibirsk. A filament volume-cusp source with normalized emittance of about 0.1 π mm mrad is used in the accelerator for the Xiamen BNCT Center [13].

Since VITA is characterized by a fast ion acceleration rate, the entrance electrostatic lens is strong. For this reason, the injected ion beam must be refocused to the entrance to the accelerator. To focus the ion beam at the entrance of the accelerator, we used a pair of magnetic lenses, which led to a noticeable spherical aberration of the ion beam and to the degradation of the normalized emittance to 0.54 π mm mrad [14]. The improvement in the quality of the ion beam was achieved by modernizing the magnetic lens: it became shorter and more powerful. Since the transport and focusing of a relatively low energy ion beam is accompanied by a spatial charge [14], a wire scanner OWS-30 (D-Pace, Nelson, BC, Canada) was used to control the position and size of the ion beam at the entrance to the accelerator.

The position and size of the ion beam in the accelerator are controlled by two pairs of video cameras overseeing the input and output diaphragms of the external accelerating electrode. Cameras register visible radiation caused by interaction of ions with residual and stripping gases, and heating of diaphragms [15].

**Figure 2:**
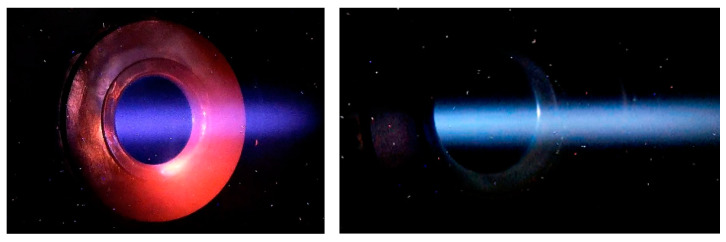
Example of video camera images (the ion beam is visible in blue, the heated diaphragm is visible in red; the image at a proton current of 9 mA is on the right).

A gas stripper is used for stripping negative hydrogen ions into protons. The stripper is a cooled tube with argon inlet through a hole in the middle. The presence of a gas stripper in a tandem accelerator is often considered a disadvantage. We managed to neutralize the disadvantages and turn the use of a gas stripper into an advantage. The additional gas flow makes it possible to visualize the beam for diagnostics of its position and size (Figure 2) and improves the high-voltage strength of the accelerating gaps [8]. Of course, additional gas injection increases the undesirable flux of secondary charged particles, but this flux is reduced to an acceptable level by improving vacuum pumping and suppressing secondary electron emission from the walls of the vacuum chamber [16]. The flux of positive argon ions formed inside the gas stripper and penetrating into the accelerating gaps turned out to be extremely low: it was 2000 times less than the proton beam current [9].

**Figure 3:**
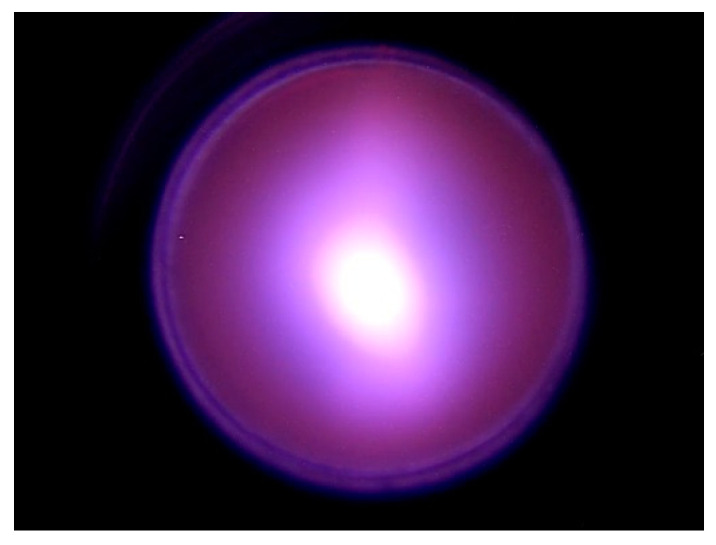
Image of video camera connected to a telescope looking into the gas stripper along the axis. Stripper hole diameter is 16 mm.

Typically, a gas stripper provides 95% conversion of negative ions to positive ones. Although a larger injection of argon leads to an increase in the proton current, the current of secondary charged particles grows much more strongly. Inside a stripping tube 16 mm in diameter, the ion beam has a size of about 5 mm and a divergence of ±2 mrad. Figure 3 shows an image of a video camera connected to a Celestron Ultima 80–45 telescope looking into the gas stripper along the axis through a cooled copper mirror. The size of the ion beam and the divergence were determined from measurements of the phase portrait of the neutral flux measured with a cooled diaphragm scanning the beam and a wire scanner located behind the scanning diaphragm and a bending magnet turned on to deflect ions.

**Figure 4:**
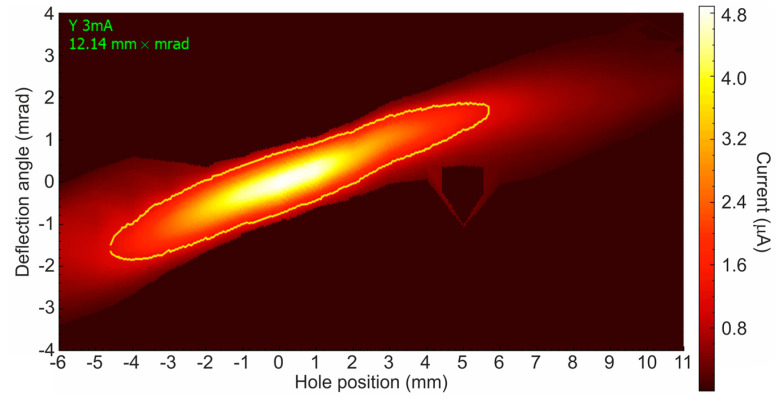
Phase portrait of a 2 MeV, 3 mA proton beam.

The proton beam obtained inside the stripper is focused by an electrostatic lens due to the penetration of the electric field through the diaphragm into the high-voltage electrode, is accelerated in the accelerating gaps, and is slightly defocused by the output electrostatic lens. At a distance of 1.86 m from the center of the accelerator, the proton beam has a characteristic size of 11 mm, a divergence of ±1.5 mrad, and a normalized emittance of 0.23 π mm mrad. An example of a phase portrait of a proton beam measured by a movable cooled diaphragm and a wire scanner OWS-30 is shown in Figure 4. It can be seen that the beam is almost perfect and there are no noticeable aberrations. Its normalized emittance is even slightly less than the emittance of a beam of negative hydrogen ions declared in [12], which is explained by the performed optimization of the ion source. The peculiarity of the proton beam, which is attractive for its transportation, lies in its sharp border—there is no beam at a distance of 2σ from the beam axis. This is due to the effect of the space charge during the transport and focusing of a beam of negative hydrogen ions and the small size of the diaphragms—10 mm in the entrance and 20 mm in the accelerating gaps.

Two cooled copper diaphragms (a hole diameter of 24 mm) with four thermocouples evenly spaced in azimuth inside are installed before the bending magnet. These diaphragms are used to optimize the production of the proton beam. By a magnetic focusing lens and a corrector in the low-energy beam path, the passage and acceleration of the ion beam are achieved such that the input diaphragm of the first accelerating electrode is heated rather weakly (diagnosed by video cameras) and the cooled diaphragms in front of the bending magnet are also heated weakly and symmetrically. Stronger focusing of negative ions makes it possible to obtain a smaller proton beam at the exit from the accelerator and with a lower divergence, but this mode is accompanied by a greater heating of the entrance diaphragm of the first accelerating electrode due to the broadening of the ion beam here. Weaker focusing of negative ions, on the contrary, does not lead to heating of the entrance diaphragm of the first accelerating electrode, but makes the proton beam more divergent. Since the accelerator operates in a wide range of ion energies and currents, as well as the type of ions—protons or deuterons, this procedure is used to optimize the production of an ion beam.

The spatial charge does not influence the transport of the proton beam [10], therefore, the proton beam can be transported relatively simply and without loss to a lithium target 10 cm in diameter in whatever position it is installed. So, with the bending magnet turned off, the size of the proton beam on the surface of the lithium target in position *C* is 20 mm, in position *D*—28 mm, *E*—38 mm (here the distance from the center of the accelerator to the target is 10.7 m). To direct the proton beam downward, a bending magnet with a decay rate of 0.5 is used, which ensures the same focusing of the proton beam in the direction along and across the magnetic field. The proton beam is transported in the vertical part when its divergence is ±8 mrad. On the surface of the lithium target at position *A*, the transverse dimension of the proton beam is 30 mm and can be increased by turning on the scanner; at position *B*, the transverse dimension of the proton beam is predicted to be 70 mm.

### 3.2. Lithium Target

“Unfortunately, while the ^7^Li(p,n)^7^Be reaction is excellent neutronically, the mechanical, chemical, and thermal properties of lithium metal make it a poor candidate for a target”, as previously was written in [17] (p. 23). Fortunately, all technical problems have been solved [18,19,20,21] and a lithium target has been developed that provides long-term stable neutron generation with relatively simple and safe maintenance.

In the design of the target, the following factors were considered: (1) The lithium layer must be thick enough to slow down protons in it only to the neutron generation threshold of 1.882 MeV. This can decrease the accompanying 0.478 MeV γ-ray flux and the temperature on the lithium surface. (2) The lithium layer must consist of pure lithium to maximize the neutron yield. The neutron yield from the lithium hydride, oxide, and fluoride is 1.43, 2, and 3.3 times lower than that from pure lithium, respectively. (3) The lithium layer must remain solid to prevent backstreaming of the lithium vapor and ^7^Be in the beam duct. (4) The substrate must be intensively cooled to maintain the lithium layer in the solid state during its heating by the powerful proton beam. (5) The substrate on which the lithium layer is deposited must be thin. This enables optimization of the moderator and placing the moderator close to the neutron generation surface. (6) The substrate must be resistant to radiation damage. (7) The target plate must be easily removable at the end of the target life. (8) All materials of the target assembly should have minimal activation by neutrons.

Lithium target 10 cm in diameter has three layers: a thin layer of pure lithium to generate neutrons in ^7^Li(p,n)^7^Be reactions; a thin layer of material totally resistant to radiation blistering; and a thin copper substrate for efficient heat removal. This target provides a stable neutron yield for a long time (at least the treatment time for 340 patients, as was measured) with an acceptably low level of contamination of the beam transport path by the inevitably formed radioactive isotope beryllium-7.

A ^7^Li(p,p’γ)^7^Li reaction cross section and 478 keV photon yield from a thick lithium target at proton energies from 0.65 MeV to 2.225 MeV were measured with high precision [22]. This knowledge made it possible to establish that at a proton energy of 2.3 MeV, the use of a thin lithium target instead of a thick one reduces undesirable 478 keV photon yield from the ^7^Li(p,p’γ)^7^Li reaction by a factor of two while maintaining the neutron flux. Additionally, this knowledge made it possible to propose and implement a method for in situ measuring of the thickness of the lithium layer [23].

### 3.3. Beam-Shaping Assembly

A Beam-Shaping Assembly (BSA) is applied to convert neutron flux into a beam of epithermal neutrons with characteristics suitable for clinical applications. The BSA consists of a magnesium fluoride moderator, a composite reflector (graphite in the front hemisphere and lead in the back), an absorber, and a filter. Monte Carlo radiation transport tools are used to design and optimize a neutron source [24,25,26,27,28], X-ray-radian- and gamma-radian-based systems [29,30]. Numerical simulations of neutron and γ-radiation transport showed that at a proton beam energy of 2.3 MeV, the BSA makes it possible to produce a neutron beam with parameters well-matching the BNCT requirements [31,32].

The flux density of epithermal neutrons is 1.04 10^9^ n/cm^2^ at a current of 10 mA while the contribution of thermal neutrons, fast neutrons, and γ-radiation is acceptably small: Φ_th_/Φ_epi_ = 1/30, *D*_fn_/Φ_epi_ = 1.25 10^−13^ Gy/cm^2^, *D*_γ_/Φ_epi_ = 1.89 10^−13^ Gy/cm^2^ [32]. Such a beam provides a dose rate in the tumor at 52.5 ppm ^10^B equal to 85 RBE Gy/h. The maximum dose ratio of tumor to normal tissue is 5.38, and the treatable depth is 7.52 cm. This BSA has been manufactured and is currently being installed in bunker 2 (see *E* position in Figure 1) for clinical trials.

A Plexiglas moderator 72 mm thick is used to produce a beam of epithermal neutrons suitable for irradiation of cell cultures and laboratory animals for development of the BNCT technique. Compared to MgF_2_ BSA, this moderator provides a higher neutron flux, but slightly worse therapeutic ratio and depth of therapy. Thus, a 2.05 MeV 1 mA proton beam provides a dose rate of 30 RBE Gy/h in cells with 40 ppm ^10^B and 6 RBE Gy/h in cells without boron.

### 3.4. Dosimetry

In Boron Neutron Capture Therapy, the total absorbed dose is the sum of four dose components with different RBE: boron dose; high-LET dose from the ^14^N(n,p)^14^C reaction (“nitrogen” dose); fast neutron dose; γ-ray dose. “The first two dose components cannot be measured in principle”, as previously was written in [1] (p. 279). We developed new approaches for measuring boron dose, nitrogen dose, and fast neutron dose.

**Figure 5:**
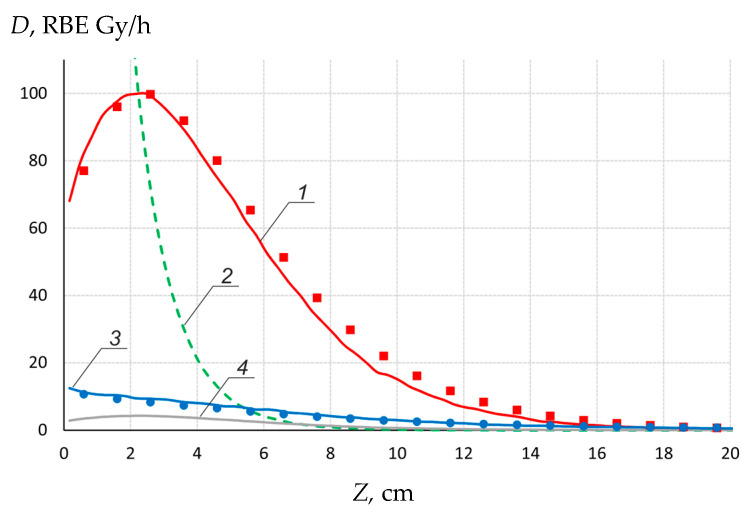
Depth distribution of boron dose (▪) and γ-ray dose (●) in a water phantom 33 × 33 × 31.5 cm^3^ close to the target at 2.05 MeV 1 mA proton beam. The calculated components of the absorbed dose: *1*—boron dose, *2*—fast neutron dose, *3*—γ-ray dose, *4*—nitrogen dose.

A small-sized neutron detector (1 mm × 1 mm in diameter) with two cast polystyrene scintillators, one of which is enriched in boron, has been developed and is being used for dosimetry of boron dose and γ-ray dose [33]. Figure 5 shows the result of measurements of the depth distribution of boron dose and γ-ray dose in a water phantom 33 × 33 × 31.5 cm^3^ close to the lithium target at 2.05 MeV 1 mA proton beam. Additionally, Figure 5 shows the calculated components of the absorbed dose.

For measuring nitrogen dose and fast neutron dose, we proposed a new approach [34]: the cell lines are irradiated by γ-radiation and mixed radiation (neutron and γ-radiation) for the same time measuring γ-ray dose. After that, comparing the doses of γ-radiation causing the same effect, for example, survival, determines the doses due to high-LET particles dose. This method was implemented [35]. It was determined that the sum of fast neutron dose and nitrogen dose is equal to 31% of the γ-ray dose for 72 mm Plexiglas moderator.

### 3.5. In Vitro and In Vivo Studies

As a result of studies carried out at the facility, it has been established that the neutron irradiation of U251 and T98G human glioma cells, pre-incubated in the medium with boron, leads to a significant suppression of their vitality [36,37]. Irradiation of immunodeficient mice with grafted human glioblastoma with pre-injection of ^10^B-enriched drugs results in their reduction or suspension [38] or their complete recovery [7]. 

### 3.6. Clinical Implementation

The developed neutron source became a prototype of the commercial neutron beam system for hospital-based BNCT manufactured by TAE Life Sciences (Foothill Ranch, CA, USA) [39]. The commercial neutron beam system consists of an evolution of the experimental VITA machine for 10 mA 2.5 MeV proton beam and solid lithium target. The improvements in the accelerator include moving a portion of the feedthrough insulator inside the high-voltage rectifier, which allows the intermediate accelerator electrodes to be directly connected to the respective rectifier section [40,41]. This modification reduces the required ceiling height for the room housing of the source and provides improved stability of the intermediate electrode potentials. TAE Life Sciences installed its first commercial neutron beam system at the new BNCT Center at Xiamen Humanity Hospital in Xiamen, P.R. China in 2020. The neutron beam system will serve as the neutron source to be incorporated with other components provided by Neuboron Medtech Ltd. (Nanjing, China), who will own and manage the full system within the BNCT Center.

In March 2021, a government decision was made to prepare for the use of BNCT in Russia [42]. The first stage of the project involves certification of the Budker Institute of Nuclear Physics (BINP) neutron source, certification of a boron delivery drug, BNCT certification, and clinical trials at the BINP neutron source. The second stage, starting next year, involves the manufacture of a hospital-based neutron source and equipping the first BNCT clinic in Russia with it.

### 3.7. Other Applications

BINP source is suitable for many other applications beyond generating epithermal neutrons for BNCT. Recently, the source was used to measure the content of hazardous impurities in boron carbide samples developed for thermonuclear fusion reactor ITER [43,44]. 

The neutron source is planned to be used for radiation tests of fibers of the laser calorimeter calibration system of the Compact Muon Solenoid electromagnetic detector developed for the High-Luminosity Large Hadron Collider in CERN [45]. To generate fast neutrons, hydrogen was replaced by deuterium, a deuteron beam with an energy of 2.1 MeV, and a current of 1.4 mA was used to generate neutrons with an average energy of 5.7 MeV. The neutron yield was 2 × 10^12^ s^−1^ [11].

The neutron source with specialized targets makes it possible to generate monochromatic γ-quanta [21] and resonance γ-quanta for the development of operational methods of explosives and drugs detection [46], α-particles for investigating promising neutron-less fusion ^11^B(p,α)αα reaction [47], and positrons through ^19^F(p,αe^+^e^−^)^16^O reaction [48].

### 3.8. Future Research

There are four main areas of research:

- Neutron source. The research carried out will be aimed at modernizing the facility in order to obtain a 2.3 MeV 10 mA dc proton beam in a long-term stable mode.
- Neutron field. The purpose of these studies is spectral and flux characterization of the high flux epithermal neutron fields produced by the neutron source using a new directional spectrometer developed by the Laboratory of Subatomic Physics and Cosmology CNRS-IN2P3, Grenoble-Alpes University (France) [49] and by detectors, methods, and standards used by the Laboratory of Micro-Irradiation, Metrology, and Neutron Dosimetry, IRSN (Cadarache, France). This characterization has never been done for an epithermal neutron field.
- Boron imaging. One of the boron imaging methods is prompt γ-ray analysis based on the fact that the neutron capture with ^10^B is accompanied by instantaneous emission of 478 keV photon. We obtained a diagnostic neutron beam exclusively in the epithermal energy range using kinematic collimation and a thin lithium target. We plan to use this beam to study the kinetics of boron accumulation in the tumor and in organs of laboratory animals in real-time regime.
- Clinical trials. As is already mentioned, the team of researchers was tasked with conducting clinical trials of the BNCT technique at the BINP neutron source in order to introduce BNCT in Russia.

## 4. Conclusions

A compact neutron source has been proposed and created at the Budker Institute of Nuclear Physics in Novosibirsk, Russia. The source comprises an original design tandem accelerator, solid lithium target, and a neutron beam shaping assembly. The neutron source produces the high neutron flux for boron neutron capture therapy, as well as for other applications. The neutron source has been used and is planned to be used for a variety of different scientific studies including clinical trials.

## Data Availability

The data presented in this study are available on request from the corresponding author.
